# Identification of Auxin-Associated Genes in Wheat Through Comparative Transcriptome Analysis and Validation of the Candidate Receptor-like Kinase Gene *TaPBL7-2B* in Arabidopsis

**DOI:** 10.3390/plants14152277

**Published:** 2025-07-24

**Authors:** Mengjie Zhang, Guangzhu Chen, Jie Cai, Yongjie Ji, Linrun Xiang, Xinhong Chen, Jun Wang

**Affiliations:** Shaanxi Key Laboratory of Genetic Engineering for Plant Breeding, College of Agronomy, Northwest A&F University, Yangling 712100, China; zhangmengjie1573@163.com (M.Z.); qpalcgz123@163.com (G.C.); 18893872883@163.com (J.C.); jiyongjie2025@163.com (Y.J.); xianglinrun@126.com (L.X.)

**Keywords:** wheat, auxin, receptor-like kinase (RLK), *TaPBL7-2B*, functional analysis

## Abstract

Auxin (IAA), a key natural signaling molecule, plays a pivotal role in regulating plant growth, development, and stress responses. Understanding its signal transduction mechanisms is crucial for improving crop yields. In this study, we conducted a comparative transcriptome analysis of wheat leaf and root tissues treated with different concentrations of IAA (0, 1, and 50 μM). Functional enrichment analysis revealed that differentially expressed genes (DEGs) exhibited tissue-specific regulatory patterns in response to auxin. Weighted Gene Co-expression Network Analysis (WGCNA) identified receptor-like kinase genes within the MEgreen module as highly correlated with auxin response, suggesting their involvement in both root and leaf regulation. Among them, *TaPBL7-2B*, a receptor-like kinase gene significantly upregulated under 50 μM IAA treatment, was selected for functional validation. Ectopic overexpression of *TaPBL7-2B* in *Arabidopsis thaliana* (Col-0) enhanced auxin sensitivity and inhibited plant growth by suppressing root development and leaf expansion. In contrast, knockout of the Arabidopsis homolog *AtPBL7* reduced auxin sensitivity and promoted both root and leaf growth. Transcriptome analysis of Col-0, the *TaPBL7-2B* overexpression line, and the *pbl7* mutant indicated that *TaPBL7-2B* primarily functions through the MAPK signaling pathway and plant hormone signal transduction pathway. Furthermore, qRT-PCR analysis of wheat varieties with differing auxin sensitivities confirmed a positive correlation between *TaPBL7-2B* expression and auxin response. In conclusion, *TaPBL7-2B* acts as a negative regulator of plant growth, affecting root development and leaf expansion in both Arabidopsis and wheat. These findings enhance our understanding of auxin signaling and provide new insights for optimizing crop architecture and productivity.

## 1. Introduction

The growth and development of higher plants, including wheat, are generally influenced by environmental conditions, phytohormone levels, and gene transcription [1,2]. Among these factors, auxin stands out as a crucial natural signaling molecule that regulates numerous processes, including cell proliferation and differentiation [3], organ formation and development [4], and stress responses [5]. Previous studies have suggested that the formation and development of the root system, as well as the accumulation of aboveground biomass during the wheat seedling stage, can significantly impact final yield [6,7]. Therefore, the role of auxin in regulating organ development, particularly in leaves and roots, is considered a promising pathway for improving wheat productivity.

Auxin-mediated changes in plant growth and development are primarily achieved by regulating cell expansion, division, and differentiation [3]. In addition, auxin influences plant organ morphogenesis through its concentration gradient distribution [4]. While moderate increases in auxin concentration can promote growth, excessively high levels may inhibit it [8]. However, the molecular mechanisms by which plant cells perceive and transmit auxin signals remain largely unclear. It is widely accepted that auxin receptors, whether extracellular or intracellular, must possess two key characteristics: the ability to bind auxin directly and to trigger downstream biological responses [9]. Several auxin receptors have been identified in plants, including Auxin Binding Protein 1 (ABP1) and its associated Transmembrane Kinases TMK1–TMK4, Transport Inhibitor Response 1 (TIR1), and its homologs Auxin Signaling F-box proteins (AFB1-AFB5), S-phase Kinase-associated Protein 2a (SKP2a), and Auxin Response Factor 3 (ARF3), also known as ETTIN or ETT [9].

The regulation of auxin signaling in plants is a complex and tightly controlled process involving the coordinated action of multiple proteins. Recent research has highlighted the crucial role of protein kinases in auxin signal transduction. For instance, the interaction between plasma membrane association of the rheostat proteins, BREVIS RADIX (BRX), and PROTEIN KINASE ASSOCIATED WITH BRX (PAX) regulates PIN-mediated auxin efflux in the protophloem sieve elements. This BRX-PAX molecular rheostat is enhanced by interaction with PHOSPHATIDYLINOSITOL-4-PHOSPHATE-5-KINASE (PIP5K) and disrupted by CLE45-BAM3-PBL34/35/36 signaling [10]. In this pathway, PBS1-like (PBL) cytoplasmic kinases, which belong to the receptor-like kinase (RLK) subfamily of the protein kinase superfamily, act as a branch of CLE45-BAM3 signaling to induce CLE45-mediated PIN patterning. Moreover, PBL34/35/36 are phosphorylated by BARELY ANY MERISTEM (BAM), affecting root apical meristem homeostasis, which is regulated by the CLAVATA3/EMBRYO SURROUNDING REGION-related (CLE) peptide signaling pathway [11].

The RLK family is the largest subgroup of plant protein kinases. As plant-specific transmembrane proteins, RLKs play essential roles in plant growth and development by transmitting extracellular ligand signals as receptors or co-receptors through reversible conformational changes [12]. A well-studied example is BRASSINOSTEROID INSENSITIVE 1 (BRI1), the primary receptor for the plant hormone brassinosteroid (BR). BRI1 mediates BR signaling to regulate cell elongation. Upon BR perception, BRI1 recruits the co-receptor BRI1-ASSOCIATED RECEPTOR KINASE 1 (BAK1) to form a receptor–co-receptor complex, which activates downstream BR signaling through phosphorylation and triggers a signal cascade [13]. Additionally, some RLKs influence the auxin signaling pathway by modulating the activity of auxin transporter PIN proteins. For instance, the auxin-regulated receptor CAMEL, a malectin-domian RLK, interacts with CANAR (canalization-related receptor-like kinase) and phosphorylates PIN, thereby regulating auxin transport and distribution [14]. Beyond their core roles in regulating plant growth, development, and hormone signaling pathways, RLKs also play significant roles in environmental adaptation. They can perceive not only abiotic stresses such as drought, high temperature, and low temperature, but also biotic stresses like pathogen infection, and they help defend against these threats by activating plant immune signaling pathways. Therefore, RLKs are crucial components in plant responses to both abiotic and biotic stresses [15].

Researchers have long studied the regulatory role of auxin in plant growth and development. However, due to the complex genetic background of hexaploid wheat (2n = 6x = 42, AABBDD), progress in understanding the molecular mechanisms underlying its morphological traits has been relatively slow. In recent years, continuous advancements in high-throughput omics technologies, coupled with significant reductions in sequencing costs, have led to the rapid accumulation of large-scale omics datasets. Comprehensive bioinformatics analyses—encompassing transcriptomics, proteomics, metabolomics, and genomics—have become essential tools for dissecting the complex traits of important crops such as wheat.

In this study, we analyzed the transcriptomic profiles of wheat leaves and roots treated with different concentrations of IAA and constructed an auxin-associated gene co-expression network. Among the identified genes, we focused on the receptor-like kinase PBL7, whose expression was significantly upregulated in both root and leaf tissues in response to auxin treatment. Previous studies have reported that the receptor-like kinase PBL7 contributes to cold stress responses in barley [16]. In addition, PBL7 plays a vital role in auxin signal transduction. During cryopreservation-induced somatic embryogenesis, long non-coding RNAs (lncRNAs) help maintain the expression of auxin signaling components, including the receptor-like kinase PBL7 and the auxin influx carrier LAX3, by acting as competing endogenous RNAs (ceRNAs) to sequester microRNAs (miRNAs). This protective mechanism preserves the integrity of the auxin signaling cascade, as evidenced by the upregulation of downstream genes. Notably, this lncRNA-mediated regulation is critical for enhancing the embryogenic potential of mature somatic embryos and promoting the formation of new embryogenic tissues [17]. These findings further highlight the indispensable role of receptor kinases in integrating stress responses with auxin signaling. We further cloned the auxin-associated receptor-like kinase gene *TaPBL7-2B* from wheat and validated its function using the Arabidopsis *pbl7* mutant, *TaPBL7-2B/pbl7* complementary lines, and *TaPBL7-2B* overexpression lines. Subsequent RNA-Seq analysis of these plants materials suggests that *TaPBL7-2B* primarily functions through the MAPK signaling pathway and the plant hormone signal transduction pathway. This study provides fundamental insights into auxin-associated receptor-like kinase genes in wheat and highlights the potential application of the auxin-associated *TaPBL7-2B* in manipulating wheat architecture through transgenic approaches.

## 2. Materials and Methods

### 2.1. Data Source and Processing

Transcriptomic and phenotypic data for wheat (*Triticum aestivum* L. cv. Chinese Spring) leaf and root samples were obtained from previous studies conducted in our laboratory [18,19]. Wheat seedlings with a uniform growth rate were selected to assess growth responses under different concentrations of indole-3-acetic acid (IAA): 0, 1, and 50 µM. Leaf and root samples were collected separately, with three biological replicates per treatment. Differentially expressed genes (DEGs) were identified using the criteria |log_2_ (fold change)| ≥ 2 and a false discovery rate (FDR) < 0.05.

### 2.2. GO and KEGG Pathway Enrichment Analysis

The biological functions of DEGs were annotated using the Gene Ontology (GO) database. GO enrichment analysis was conducted using the GOseq package, with a significance threshold of *p* value < 0.05 [20]. In addition, DEG-associated metabolic and signal transduction pathways were analyzed on the basis of the Kyoto Encyclopedia of Genes and Genomes (KEGG) database. KEGG enrichment analysis was performed using the clusterProfiler package (version 3.12.0) [21]. The enrichment results were visualized using an online platform (https://www.bioinformatics.com.cn (accessed on 12 July 2023)).

### 2.3. Co-Expression Network Analysis and Key Module Identification

DEGs from the transcriptomic data of 15 samples, along with phenotypic data from IAA treatments, were subjected to Weighted Gene Co-expression Network Analysis (WGCNA) using the R package (version 4.1.2) [22]. A symmetric adjacency matrix was constructed from gene expression data, where each value represents the connection strength between a pair of genes. This matrix was generated on the basis of pairwise gene correlation coefficients and transformed using a soft-threshold power to approximate a scale-free topology. The Topological Overlap Measure (TOM) was applied to quantify gene interconnectivity within the network.

Gene modules were identified using hierarchical clustering and further refined using the dynamic tree cut algorithm. Module membership (MM) was calculated to quantify the correlation between individual genes and their corresponding modules. In addition, phenotypic data were integrated to assess module–trait relationships by evaluating Gene Significance (GS) and Module Significance (MS), thereby identifying modules strongly associated with IAA-responsive traits.

### 2.4. Plant Materials and Growth Conditions

The experimental design for wheat samples has been described in our previous studies [18,19]. Briefly, wheat seeds were surface-sterilized with a 1% NaClO solution for 15 min, rinsed three times with distilled water, and kept in the dark at 4 °C for 2 days. The seeds were then transferred to a growth chamber under a 16/8 h day/night photoperiod at 24/17 °C (day/night). After 1 day, 30 healthy seedlings with approximately equal root lengths (indicating uniform growth) were selected and transferred to Hoagland’s solution containing different concentrations of IAA (0, 1, and 50 μM). After 1 week, leaf and root samples from 3–10 individual plants in each replicate were collected and pooled as one sample for transcriptomic and qRT-PCR analyses. The wheat varieties used in this study were sourced from our laboratory’s germplasm collection. For Arabidopsis, seeds were surface-sterilized and sown on plates containing 1/2 MS medium supplemented with 1% sucrose, 0.8% agar, and varying concentrations of IAA (0, 1, and 50 μM). The plates were incubated in darkness at 4 °C for 2 d and then transferred into a growth chamber (16/8 h day/night photoperiod under 24/17 °C day/night temperatures). Light intensity in the chamber was maintained at approximately 100 μmol m^−2^ s^−1^. *Arabidopsis thaliana* ecotype Columbia-0 (Col-0) and the T-DNA insertion mutant *pbl7* (SALK_114130) were obtained from Arashare (https://www.arashare.cn (accessed on 6 November 2021)), and their genetic backgrounds were confirmed by PCR. Specific primers were designed using the T-DNA primer design tool (http://signal.salk.edu/tdnaprimers.2.m.html (accessed on 15 December 2022)), and the sequences are listed in Table S1.

### 2.5. Transgenic Plant Construction

The full-length CDS of the *TaPBL7-2B* gene was cloned from the wheat variety Chinese Spring via PCR amplification using PrimeSTAR^®^ HS DNA Polymerase (TAKARA, Dalian, China). The amplified product was purified and ligated into the pRI201-AN-GUS overexpression vector (TAKARA, Dalian, China) through homologous recombination using the OK Clon DNA Ligation Kit II (Accurate Biology, Changsha, China). After sequence verification to ensure the *TaPBL7-2B* CDS was 100% correct, the recombinant plasmid was introduced into *Agrobacterium tumefaciens* strain GV3101 and transformed into *Arabidopsis thaliana* using the floral dipping method [23]. Transgenic plants were screened on plates containing 1/2 MS medium supplemented with 1% sucrose, 0.8% agar, and 50 μg/mL kanamycin. At least two independent *TaPBL7-2B* overexpression lines (OE-1 and OE-2) and two *TaPBL7-2B/pbl7* complementary lines (*TaPBL7-2B/pbl7-1* and *TaPBL7-2B/pbl7-2*) were obtained and selected for subsequent phenotypic and functional analyses.

### 2.6. IAA Treatments and Phenotypic Analysis

To measure the primary root length of Arabidopsis, seeds were uniformly sown on plates containing 1/2 MS medium supplemented with 1% sucrose and 0.8% agar (control group). For the treatment group, IAA was added to a final concentration of 10 nM. After incubating at 4 °C for 2 days, both groups were transferred to a growth chamber and cultivated vertically for 7 days.

To assess the leaf blade area of Arabidopsis, seeds were first incubated at 4 °C for 2 days on plates containing 1/2 MS medium supplemented with 1% sucrose and 0.8% agar, then transferred to a growth chamber and grown horizontally for 7 days. Seedlings exhibiting uniform growth were selected and transplanted into substrate-filled pots. These seedlings were cultivated under controlled conditions for another 7 days, during which leaves were sprayed with 10 µM IAA three times daily. The control group was treated with equal volumes of double-distilled water under identical conditions.

For measuring root and leaf lengths in wheat seedlings, seeds were evenly distributed on petri dishes lined with moist filter paper and incubated at 4 °C for 2 days. They were then transferred to a growth chamber for 24 h. Uniform seedlings were selected and transferred to a liquid culture system. The control group was grown in Hoagland’s solution, while the treatment group was grown in Hoagland’s solution with a final concentration of 50 µM IAA, both for 7 days.

After treatment, all plant materials were photographed, and the primary root and leaf lengths were measured using ImageJ (version 1.53n). The data were analyzed statistically using GraphPad Prism (version 9.0.0).

### 2.7. Quantitative Real-Time PCR Analysis

Total RNA was extracted from plant samples using the MiniBEST Plant RNA Extraction Kit (TAKARA, Dalian, China). First-strand complementary DNA (cDNA) synthesis was performed using the PrimeScript™ II 1st Strand cDNA Synthesis Kit (TAKARA, Dalian, China). Gene-specific primers were designed using Primer Premier 6.0. Quantitative real-time PCR (qRT-PCR) was performed using the ChamQ SYBR qPCR Master Mix (Vazyme, Nanjing, China). The *AtACTIN* and *TaACTIN* genes from Arabidopsis and wheat served as reference genes to normalize expression levels. Relative gene expression was calculated using the 2^−ΔΔCT^ method [24]. Each reaction was conducted with a minimum of three replicates. Primer sequences used in this study are listed in Table S1.

### 2.8. Library Construction and RNA Sequencing

Total RNA was extracted from Arabidopsis samples using the MiniBEST Plant RNA Extraction Kit (TAKARA, Dalian, China). RNA purity was assessed using a NanoDrop™ One/OneC spectrophotometer (Thermo Fisher Scientific, Waltham, MA, USA), and the concentration was quantified using the Qubit™ RNA HS Assay Kit (Thermo Fisher Scientific, Waltham, MA, USA). RNA integrity was evaluated with the Agilent 4200 TapeStation system.

To construct the sequencing library, mRNA with a poly(A) structure was captured using magnetic beads coated with Oligo(dT). The first strand of cDNA was synthesized from fragmented mRNA using the M-MuLV reverse transcriptase system and random primers. The RNA strand was subsequently degraded with RNase H, and the second strand of cDNA was synthesized using dNTPs in the DNA polymerase I system. The double-stranded cDNA was then purified, and end repair was conducted, followed by the addition of an A tail and ligation of the sequencing adapter. cDNA fragments of approximately 200 bp were selected using AMPure XP beads, and the PCR products were amplified and purified again using AMPure XP beads to obtain the final sequencing library.

Once the library was successfully constructed, its concentration was quantified using Kapa qPCR quantification, and the fragment size was assessed using the Agilent 4200 TapeStation. Finally, sequencing was performed using the Illumina PE150/MGI/T7 platform.

Quality control and preprocessing of the raw sequencing data were conducted with the fastp tool. Alignment of the filtered reads to the reference genome (*Arabidopsis thaliana*, TAIR10) was performed using STAR (https://github.com/alexdobin/STAR (accessed on 10 November 2023)), and transcripts were assembled with the StringTie method. Gene expression levels were estimated using the Fragments Per Kilobase of exon model per Million mapped fragments (FPKM) method.

## 3. Results

### 3.1. Effect of IAA Treatment on Wheat Seedlings

**Figure 1:**
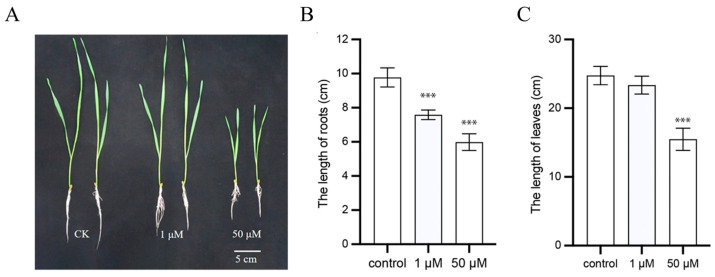
Phenotypic effects of IAA treatment on wheat seedlings. (**A**) Seven-day-old wheat seedlings grown in nutrient solutions containing 0, 1, or 50 µM IAA. (**B**) Quantification of primary root lengths corresponding to (**A**). (**C**) Quantification of leaf lengths corresponding to (**A**). Error bars represent the standard deviation (SD) of three biological replicates. Asterisks indicate significant differences compared with the control group determined by Student’s *t*-test (*** *p* < 0.001).

Wheat seedlings with uniform growth were cultured in nutrient solutions containing 0, 1, or 50 µM IAA for seven days, after which, root and leaf lengths were measured. According to our previous studies, compared with the control group, seedlings treated with 1 µM IAA exhibited a significant reduction in root length, while no significant difference was observed in leaf length. In contrast, treatment with 50 µM IAA resulted in a significant reduction in both root and leaf lengths (Figure 1). On the basis of these results, samples for subsequent comparative analysis in this study were selected as follows: (1) those exhibiting significant differences in root length under 1 µM and 50 µM IAA treatments compared with the control, and (2) those showing significant differences in leaf length under the 50 µM IAA treatment.

### 3.2. Comparative Analysis of Transcriptome Data Across Wheat Tissues

**Figure 2:**
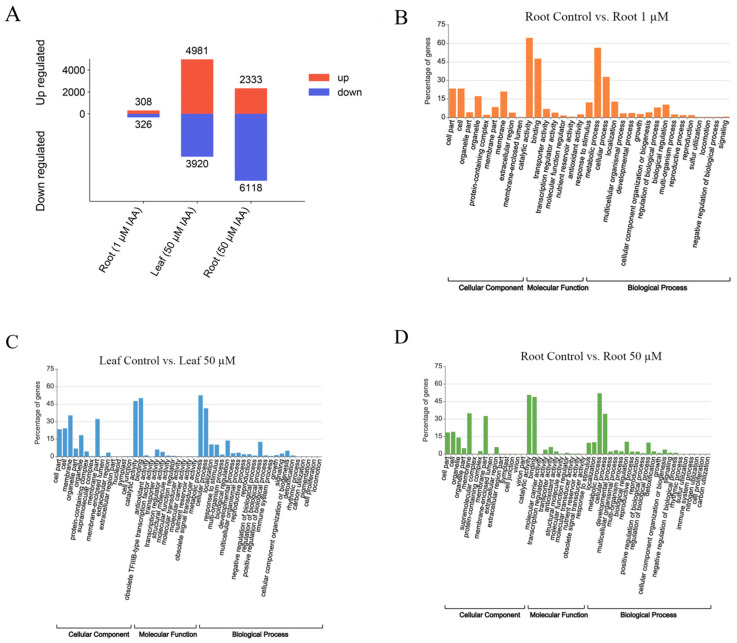
Differentially expressed gene (DEG) analysis and Gene Ontology (GO) enrichment in wheat leaves and roots. (**A**) Total number of upregulated and downregulated DEGs identified in leaf and root tissues under IAA treatment. (**B**) GO enrichment analysis of DEGs in roots (control vs. 1 µM IAA). (**C**) GO enrichment analysis of DEGs in leaves (control vs. 50 µM IAA). (**D**) GO enrichment analysis of DEGs in roots (control vs. 50 µM IAA).

Under 1 µM IAA treatment, 634 differentially expressed genes (DEGs) were identified in root samples, including 308 upregulated and 326 downregulated genes. Under 50 µM IAA treatment, 8901 and 8451 DEGs were detected in leaf and root samples, respectively (Figure 2A). Notably, 55.96% of DEGs in leaves exhibited increased expression after 50 µM IAA treatment, whereas 72.39% of DEGs in roots showed decreased expression. These contrasting patterns highlight the tissue-specific regulatory adaptions of wheat to auxin, even under the same treatment conditions.

Gene Ontology (GO) enrichment analysis revealed shared functional categories among DEGs in the treatment groups. Compared with the control, significantly enriched GO terms included catalytic activity, binding, metabolic process, cellular process, and cell parts. These results suggest that auxin treatment broadly activates catalytic functions and modulates metabolic processes in both leaves and roots (Figure 2B–D).

**Figure 3:**
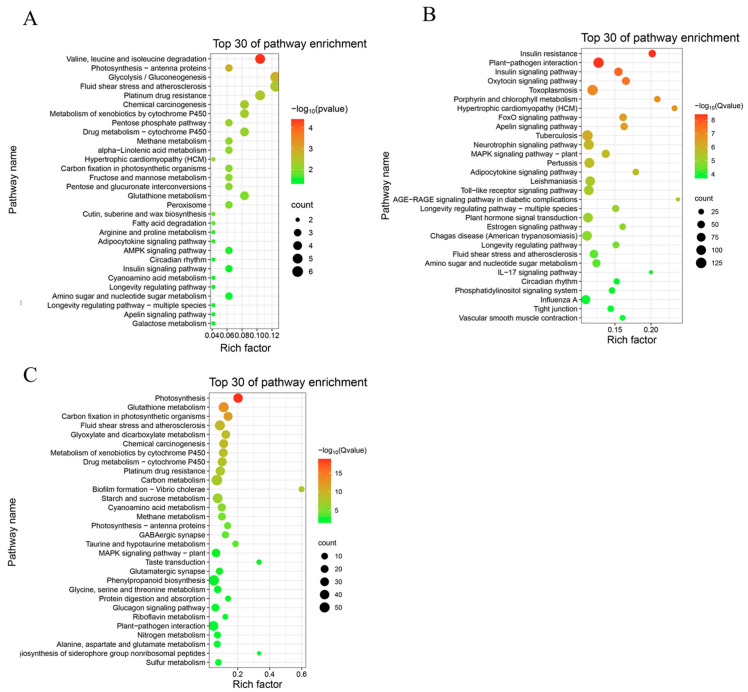
Scatter plot displaying the top 30 significantly enriched KEGG pathways identified among differentially expressed genes (DEGs). (**A**) KEGG enrichment analysis of DEGs in roots (control vs. 1 µM IAA). (**B**) KEGG enrichment analysis of DEGs in leaves (control vs. 50 µM IAA). (**C**) KEGG enrichment analysis of DEGs in roots (control vs. 50 µM IAA). The rich factor represents the ratio of the number of DEGs annotated in a given pathway to the total number of genes annotated in that pathway. Dot color corresponds to the −log_10_ (Q value), and dot size indicates the number of DEGs annotated to each pathway.

In contrast, Kyoto Encyclopedia of Genes and Genomes (KEGG) pathway enrichment analysis revealed distinct tissue-specific regulatory patterns. Compared with the control, root samples treated with 1 µM IAA were enriched in 42 pathways, while those treated with 50 µM IAA were enriched in 36 pathways. Leaf samples treated with 50 µM IAA showed enrichment in 128 pathways. In leaves, significantly enriched pathways included plant–pathogen interaction, plant hormone signal transduction, and MAPK signaling pathway—plant, likely reflecting auxin-regulated defense mechanisms and signal transduction processes. In contrast, root-enriched pathways included carbon metabolism, phenylpropanoid biosynthesis, and glutathione metabolism, suggesting roles in energy metabolism, secondary metabolite production, and oxidative stress responses (Figure 3A–C).

### 3.3. Co-Expression Network Construction and Hub Gene Identification

**Figure 4:**
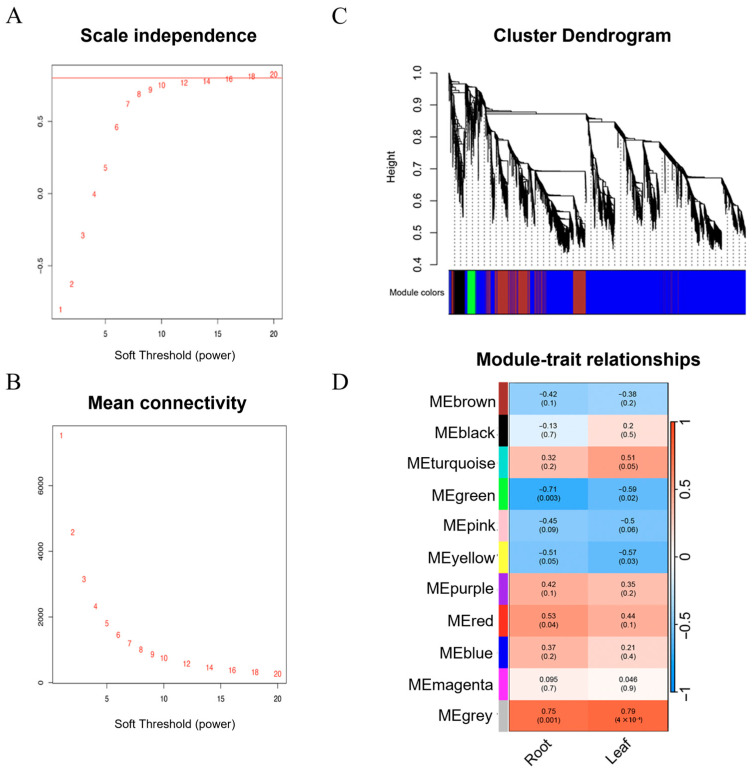
Construction of the weighted gene co-expression network and module–trait relationships. (**A**,**B**) Determination of the soft-thresholding power (*β*) based on scale-free topology criteria. The red line in (A) represents the scale-free topology fit index, with R^2^ = 0.80. (**C**) Hierarchical clustering and module assignment of genes using the dynamic tree cut method. (**D**) Heatmap showing the correlation between module eigengenes and phenotypic traits. Each cell displays the correlation coefficient (top) and the corresponding *p*-value (bottom). Red and blue colors indicate positive and negative correlations, respectively.

To identify hub genes and regulatory networks responsive to auxin in wheat leaves and roots, Weighted Gene Co-expression Network Analysis (WGCNA) was performed on 16,325 differentially expressed genes (DEGs) from three experimental groups. Based on the scale-free topology model fit index and average connectivity, a soft-thresholding power of *β* = 18 was selected to construct the weighted network (Figure 4A,B). At this *β* value, the scale-free topology fit index R^2^ reached 0.80, indicating good scale independence and an accurate representation of the gene co-expression relationships.

The network was then partitioned into modules using the dynamic tree cut method with a mergeCutHeight of 0.25 and a minimum module size of 30 genes (Figure 4C). Ultimately, the 16,325 DEGs were clustered into 11 modules (Figure 4D). Each module was assigned a color name: the grey module contained 215 unassigned genes, the smallest module (MEpurple) comprised 40 genes, and the largest module (MEturquoise) included 4620 genes.

To assess the association between modules and auxin-mediated traits in wheat leaves and roots, correlations between module eigengenes and phenotypic data were calculated. Among the 11 modules, the MEgreen module (containing 1255 DEGs) exhibited the strongest correlation with root length (*r* = −0.71, *p* = 0.003) and leaf length (*r* = −0.59, *p* = 0.02). These results suggest that genes within the MEgreen module may play pivotal roles in mediating auxin adaption in both roots and leaves.

### 3.4. qRT-PCR Verification of RLK Gene Expression in the MEgreen Module

**Figure 5:**
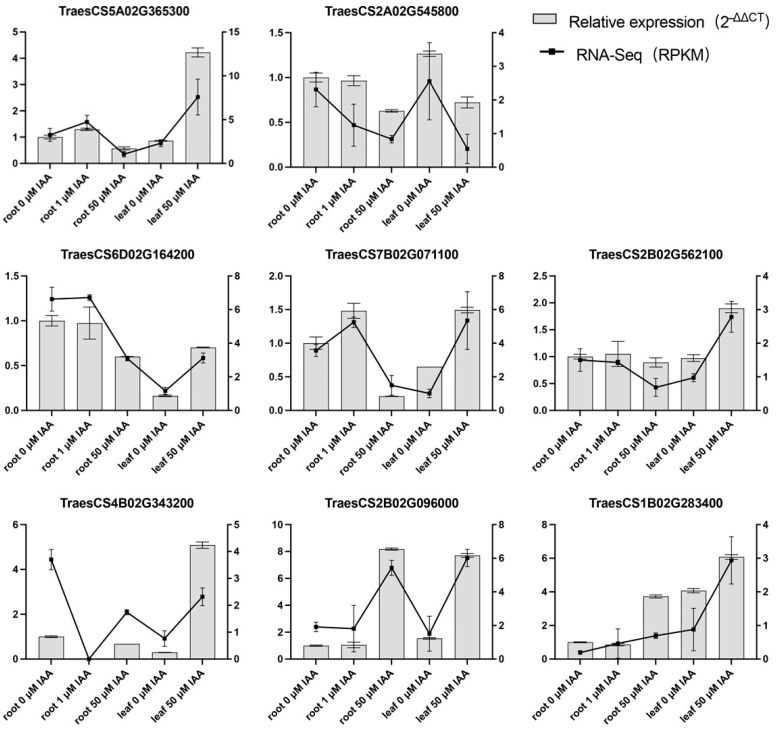
Gene expression patterns of selected genes in wheat root and leaf tissues under different auxin concentrations. RT-qPCR was performed with three biological replicates, and expression levels in the control group were normalized to 1.0. Bars represent relative qRT-PCR expression levels (left y-axis), while lines indicate the corresponding RNA-Seq expression levels (right y-axis). Data are presented as the mean ± standard deviation (*n* = 3).

The MEgreen module showed the highest correlations with leaf length (*r* = −0.59, *p* = 0.02) and root length (*r* = −0.71, *p* = 0.003), suggesting that genes within this module are likely involved in auxin-associated adaptive transcriptional regulation in both leaves and roots. Among these genes, receptor-like kinases (RLKs) attracted particular attention, as RLKs have been reported to participate in auxin-mediated signaling pathways and regulate plant growth across various tissues and developmental stages [25]. Therefore, we selected eight RLK genes for validation by quantitative real-time PCR (qRT-PCR). The expression patterns observed by qRT-PCR were generally consistent with the RNA-Seq data (Figure 5). Notably, TraesCS2B02G096000 showed significant auxin-induced upregulation in both leaf and root tissues and was chosen as a candidate gene for further functional analysis.

### 3.5. Identification of the pbl7 T-DNA Insertion Mutant and Generation of TaPBL7-2B Transgenic Arabidopsis Lines

**Figure 6:**
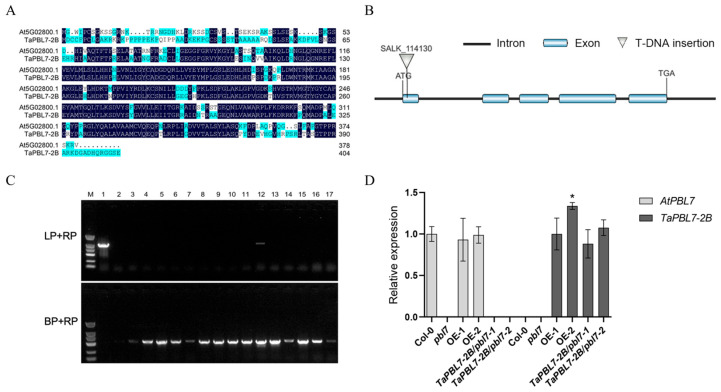
Multiple sequence alignment, identification of T-DNA insertion mutants, and generation of transgenic Arabidopsis lines. (**A**) Amino acid sequence alignment between *TaPBL7-2B* and its Arabidopsis homolog *AtPBL7*, The highly conserved amino acids are shown in black. (**B**) Schematic representation of the T-DNA insertion site in the *AtPBL7* gene. (**C**) PCR-based genotyping of *pbl7* mutants using specific primer pairs (LP + RP and BP + RP). Lane 1: Col-0; Lanes 2–17: homozygous mutant identification. (**D**) qRT-PCR analysis of *AtPBL7* and *TaPBL7-2B* expression levels in Col-0, *TaPBL7-2B*/*pbl7* complementary lines, and *TaPBL7-2B* overexpression lines. Asterisk indicates significant differences compared with the OE-1 (expression levels are normalized to 1.0) determined by Student’s *t*-test (* *p* < 0.05).

Multiple sequence alignment revealed that TraesCS2B02G096000 shares 71.4% sequence similarity with its Arabidopsis homolog AtPBL7 (At5G02800.1) (Figure 6A). On the basis of its chromosomal location, this gene was designated *TaPBL7-2B*. To investigate its biological function, *pbl7* T-DNA insertion mutant seeds were ordered from Arashare (https://www.arashare.cn). The T-DNA insertion site is located within an exon of AtPBL7, likely resulting in a loss of gene function (Figure 6B). Homozygous T-DNA insertion *pbl7* mutants were identified by three-primer PCR amplification (Figure 6C). *TaPBL7-2B* was overexpressed in both the homozygous *pbl7* mutant and wild-type Col-0 under the control of the constitutive 35S promoter, generating *TaPBL7-2B*/*pbl7* complementary lines and *TaPBL7-2B* overexpression lines. The expression levels of *AtPBL7* and *TaPBL7-2B* in various plant materials were assessed by qRT-PCR (Figure 6D).

### 3.6. TaPBL7-2B Acts as a Negative Regulator of Auxin Adaption

**Figure 7:**
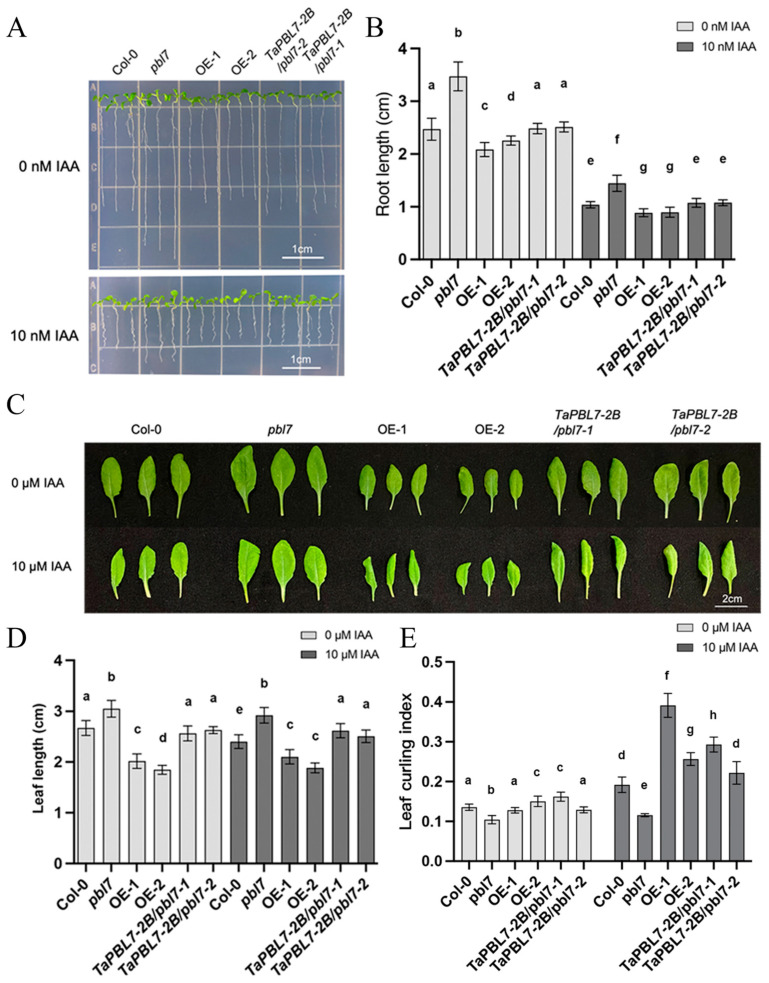
Phenotypic analysis of the Arabidopsis ecotype (Col-0), *pbl7* mutant, overexpression (OE), and *TaPBL7-2B/pbl7* complementary lines. (**A**) Primary root lengths of seedlings grown on media containing 0 nM or 10 nM IAA for 7 days. Scale bar = 1 cm. (**B**) Quantification of primary root lengths shown in (**A**). (**C**) Rosette leaves sprayed with double-distilled water or 10 μM IAA for 7 consecutive days following 7 days of normal growth after germination. Scale bar = 2 cm. (**D**) Quantification of leaf length shown in (**C**). (**E**) Quantification of the leaf curling index based on (**C**). All statistical data were obtained from at least 15 plants per line. Each dot represents an individual plant. Error bars represent the standard deviation (SD). Different letters above the bars indicate statistically significant differences compared with the Col-0 control determined by Student’s *t*-test.

To investigate the role of *TaPBL7-2B* in plant morphogenesis, we measured the primary root lengths of the *pbl7* mutant, *TaPBL7-2B*/*pbl7* complementary lines, and *TaPBL7-2B* overexpression lines grown on 1/2 MS medium supplemented with 1% sucrose and 0.8% agar. Under normal growth conditions, the *pbl7* mutant seedlings showed the longest primary root. In contrast, the primary root lengths of *TaPBL7-2B* overexpression lines were significantly reduced, indicating that *TaPBL7-2B* overexpression may inhibit primary root growth in Arabidopsis (Figure 7A,B). Moreover, under 10 nM IAA treatment, primary root growth was suppressed in all Arabidopsis plants. However, the *pbl7* mutant maintained relatively longer roots compared with Col-0 and the transgenic lines, further suggesting that *TaPBL7-2B* negatively regulates root development in adaption to auxin. Notably, constitutive expression of *TaPBL7-2B* driven by the 35S promoter partially restored the growth and development of the *pbl7* mutant to levels similar to those of Col-0.

Additionally, *TaPBL7-2B* overexpression inhibited leaf expansion. Under normal conditions, overexpression lines showed growth suppression, and after treatment with 10 μM IAA, their leaves exhibited severe curling. In contrast, the *pbl7* mutant showed nosignificant growth inhibition under either normal or auxin-treated conditions (Figure 7C–E). Overall, *TaPBL7-2B* plays a negative regulatory role in Arabidopsis growth by modulating key developmental processes such as root elongation and leaf expansion, particularly in adaption to auxin.

### 3.7. Investigation of the Transcriptional Changes of TaPBL7-2B Using RNA-Seq

To explore the potential auxin-associated transcriptional changes of *TaPBL7-2B*, RNA-Seq analysis was performed on *Arabidopsis* ecotype Col-0, the *TaPBL7-2B* heterologous overexpression line OE-1, and the *pbl7* mutant. A total of 208 and 134 differentially expressed genes (DEGs) were identified in the overexpression line and mutant, respectively. Under normal growth conditions, overexpression of *TaPBL7-2B* led to upregulation of 64.90% of DEGs, whereas loss of *AtPBL7* function in the *pbl7* mutant resulted in downregulation of 74.63% of DEGs. These results suggest that *TaPBL7-2B* may promote plant growth by upregulating downstream gene expression, highlighting its regulatory role in modulating transcriptional programs.

**Figure 8:**
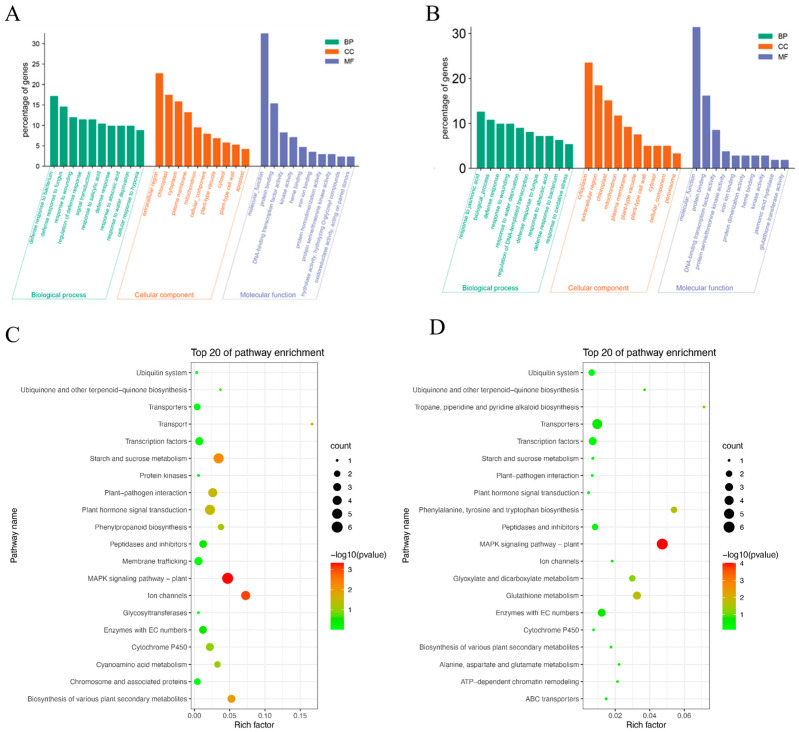
Functional enrichment analysis of differentially expressed genes (DEGs) in the *TaPBL7-2B* overexpression line 1 (OE-1) and the *pbl7* mutant. (**A**) Histogram showing Gene Ontology (GO) term enrichment of DEGs in OE-1. (**B**) Histogram showing GO term enrichment of DEGs in *pbl7*. (**C**) KEGG pathway enrichment analysis of DEGs in OE-1 displayed as a scatter plot. (**D**) KEGG pathway enrichment analysis of DEGs in *pbl7* displayed as a scatter plot.

Gene Ontology (GO) enrichment analysis revealed that DEGs from both the overexpression line and the mutant were annotated across the three major GO categories: Biological Process (BP), Cellular Component (CC), and Molecular Function (MF). In the overexpression line, 63.94% of DEGs were annotated to 435 GO terms, while in the mutant, 62.69% of DEGs were annotated to 324 GO terms. The top 10 most enriched terms in each category are presented in Figure 8A (OE),B (mutant). In the BP category, DEGs were primarily associated with defense response, hormone-related functions, and signal transduction, indicating that *TaPBL7-2B* may influence defense mechanisms and hormonal regulation. In the CC category, DEGs were enriched in the cytoplasm, chloroplast, and extracellular regions, suggesting a role in energy metabolism and intercellular communication. In the MF category, DEGs were associated with protein binding, DNA-binding transcription factor activity, and kinase activity—functions commonly involved in gene regulation, signaling, and cell-to-cell communication. These findings further support the role of *TaPBL7-2B* in orchestrating complex biological processes in plants.

**Figure 9:**
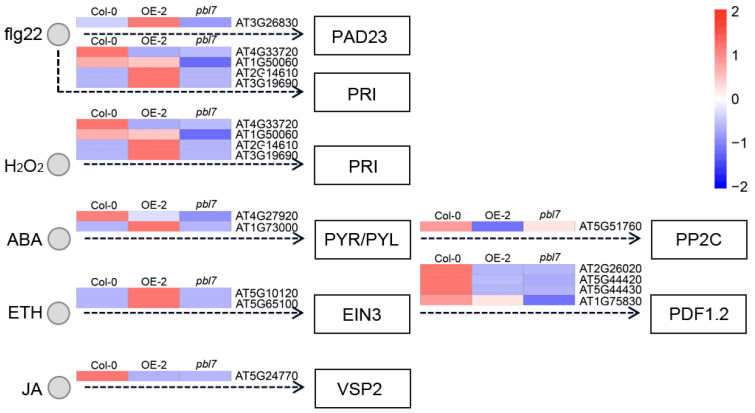
Heatmap of differentially expressed genes (DEGs) involved in the plant MAPK signaling pathway.

**Figure 10:**
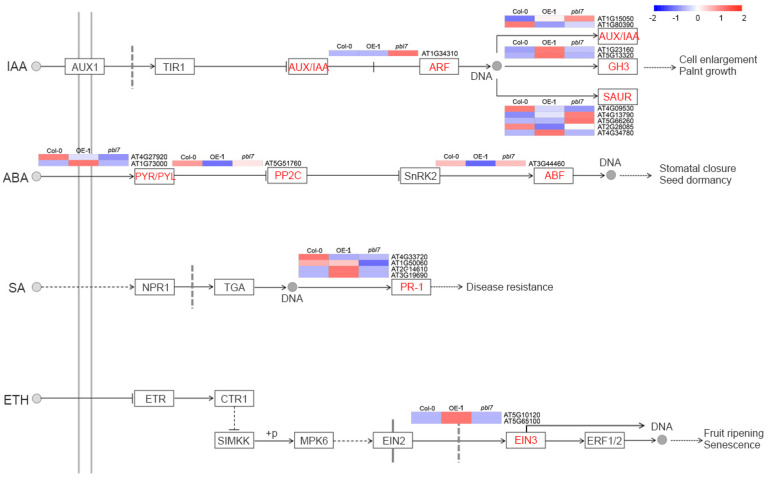
Heatmap of differentially expressed genes (DEGs) involved in the plant hormone signaling pathway.

KEGG pathway enrichment analysis showed that DEGs in both the overexpression line and the mutant were significantly enriched in various metabolic and signaling pathways. The top 20 enriched pathways are shown in Figure 8C,D. In the overexpression line, DEGs were predominantly enriched in the plant MAPK signaling pathway, starch and sucrose metabolism, and plant hormone signal transduction. In contrast, DEGs in the mutant were enriched in the MAPK signaling pathway, transporter activity, transcription factor networks, and glutathione metabolism. Notably, the plant MAPK signaling pathway was significantly enriched in both genotypes, suggesting that *TaPBL7-2B* may regulate growth and development via this conserved signaling route (Figure 9). To investigate how *TaPBL7-2B* functions in these pathways, The heatmaps were drawn with notations referenced to KEGG Pathway Map Viewer (https://www.kegg.jp/kegg/document/help_pathway.html (accessed on 29 June 2025)). Within the MAPK signaling pathway, 15 DEGs were shared between the overexpression line and mutant. These genes are involved in ethylene (ETH), jasmonic acid (JA), abscisic acid (ABA), and processes related to plant immunity, such as flg22 and H_2_O_2_ responses. In the overexpression line, these genes were consistently upregulated, implying that *TaPBL7-2B* positively regulates these stress and hormone signaling pathways, thereby contributing to enhanced physiological responses and plant development. Additionally, plant hormone signal transduction pathways were closely examined. A total of 20 DEGs in both the overexpression line and the mutant were involved in the salicylic acid (SA), ABA, auxin (IAA), and ETH pathways (Figure 10). In particular, genes involved in the auxin signaling pathway, which is closely associated with cell expansion and plant growth, displayed notable expression differences. Compared with the *TaPBL7-2B* overexpression line, the *pbl7* mutant exhibited upregulation of early auxin-associated genes, including AUX/IAA (AT1G15050, AT1G80390), ARF (AT1G34310), and SAUR (AT4G09530, AT4G13790, AT5G66260, AT2G28085, AT4G34780). In contrast, GH3 family genes (AT1G23160, AT5G13320), which encode enzymes that conjugate IAA to amino acids and thereby reduce free auxin levels, were downregulated in the *pbl7* mutant. These transcriptional patterns may reflect altered auxin homeostasis or long-term adaptation, rather than classical transient responses. They are consistent with the observed phenotypes: enhanced root and leaf growth in the *pbl7* mutant, and growth inhibition in *TaPBL7-2B* overexpression lines. Collectively, these results indicate that *TaPBL7-2B* regulates plant growth and development by modulating gene expression through hormone and MAPK signaling pathways, particularly influencing auxin signaling and stress responses.

### 3.8. Expression of the TaPBL7-2B Gene in Different Wheat Varieties

**Figure 11:**
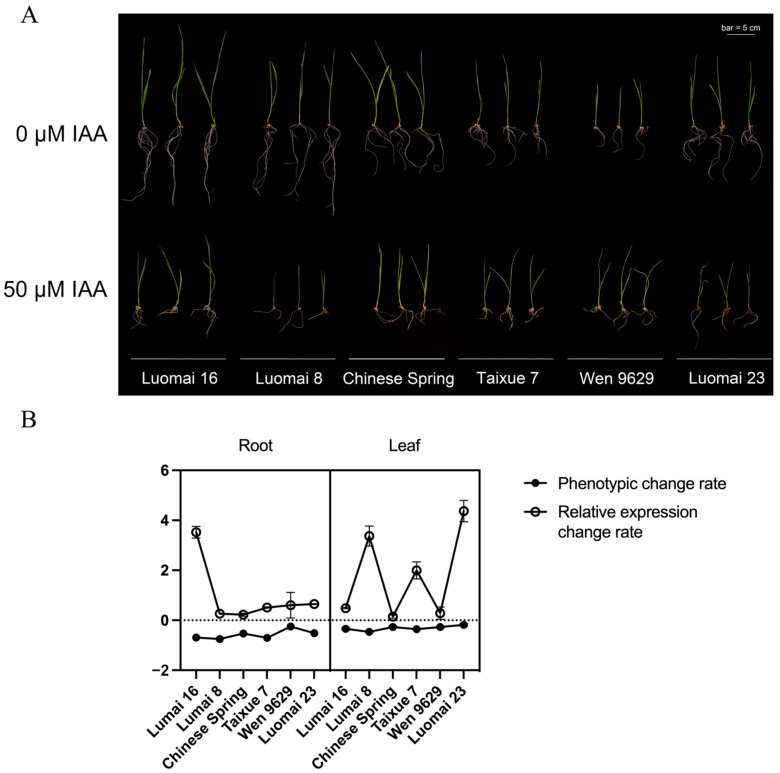
Phenotypic response and expression levels of *TaPBL7-2B* in six wheat varieties under IAA treatment. (**A**) Phenotypes of wheat seedlings grown for 7 days in Hoagland’s solution containing 0 μM or 50 μM IAA. Scale bar = 5 cm. (**B**) Rate of change in root and leaf lengths, as well as *TaPBL7-2B* expression levels, in six wheat varieties following IAA treatment. Gene expression was quantified by qRT-PCR using *TaACTIN* as the internal reference gene. Relative expression levels were normalized to the control variety Chinese Spring. The rate of change was calculated as (final value–initial value)/initial value. Error bars represent the standard deviation (SD) among biological replicates.

To further investigate the role of *TaPBL7-2B* in wheat, six wheat varieties preserved in the laboratory were grown hydroponically in Hoagland’s solution, with or without 50 μM IAA supplementation. Root and leaf lengths were measured to assess auxin sensitivity. Following treatment with 50 μM IAA, root and leaf lengths of Luomai 8 and Luomai 16 were significantly reduced, indicating strong auxin sensitivity. In contrast, Luomai 23 and Wen 9629 exhibited only minor phenotypic changes, suggesting low sensitivity to auxin. Chinese Spring and Taixue 7 displayed intermediate responses (Figure 11A). In addition, the expression levels of *TaPBL7-2B* were also checked by qRT-PCR. The results indicated that the expression of *TaPBL7-2B* was significantly upregulated in Lumai 8 and Lumai 16, while the other cultivars showed relatively minor changes (Figure 11B). These results suggest a positive correlation between *TaPBL7-2B* expression and auxin sensitivity in wheat, implying that *TaPBL7-2B* may play a regulatory role in mediating auxin-associated growth.

## 4. Discussion

### 4.1. Differential Expression Patterns in Response to the Same Concentration of Auxin in Wheat Leaves and Roots

Auxin, a crucial endogenous hormone in plants, is ubiquitous across various tissues and developmental stages, finely regulating plant growth and development. Auxin signal transduction and the associated transcriptional changes involve a complex regulatory network that modulates plant morphogenesis and stress responses by altering the transcriptional levels of downstream genes, thereby enabling plants to adapt to diverse environmental conditions. For example, overexpression of *OsIAA18* in *Oryza sativa* upregulates genes involved in the abscisic acid (ABA) signaling pathway, proline synthesis, and reactive oxygen species (ROS) scavenging enzymes, thereby enhancing tolerance to drought and salt stress [26]. Similarly, overexpression of *TaIAA15-1A* in *Brachypodium distachyon* significantly improves drought tolerance through regulation of the ABA signaling pathway [27]. In addition to transcriptional regulation, auxin also plays a key role in cell morphogenesis through rapid, non-transcriptional responses. According to the acid growth theory, auxin activates H^+^-ATPase pumps in the plasma membrane, which release protons (H^+^) into the apoplast (cell wall space), creating an acidic environment that promotes the activity of cell wall-loosening enzymes, thereby enabling rapid cell expansion [28]. This mechanism provides a theoretical foundation for improving crop architecture. Overall, elucidating the auxin regulatory network and leveraging it to optimize wheat architecture and enhance stress tolerance through transgenic approaches represents a promising direction for wheat breeding.

In this study, when wheat leaves and roots were treated with the same concentration of auxin, a similar number of genes exhibited altered expression levels; however, the nature of these changes differed significantly between tissues. Transcriptome analysis revealed that auxin treatment primarily caused downregulation of gene expression in roots, whereas in leaves, it mainly led to upregulation. The observed downregulation of gene expression in roots likely reflects the geometric and structural constraints within root cell files, which may restrict cellular expansion and contribute to auxin-induced root growth inhibition. This spatial limitation also appears to suppress the unidirectional auxin transport from the shoot apical meristem (SAM) to the root over time. In contrast, leaves maintain active and potentially enhanced multidirectional auxin transport, which may contribute to the observed upregulation of gene expression. Gene Ontology (GO) and Kyoto Encyclopedia of Genes and Genomes (KEGG) enrichment analyses further demonstrated that the differentially expressed genes (DEGs) in leaves and roots were enriched in distinct functional categories. Specifically, DEGs in leaves were predominantly involved in signal transduction pathways, while those in roots were mainly enriched in metabolic pathways.

However, it is important to note that these observations were made at a single late time point (168 h after treatment), and therefore the transcriptional changes detected likely reflect long-term transcriptional adaptations rather than rapid or primary auxin responses. Classical early auxin-responsive genes, such as AUX/IAA, ARF, and SAUR, are typically induced rapidly after auxin treatment and then return to basal expression levels. The sustained upregulation of these genes after 7 days of IAA treatment may reflect long-term adaptation, hormonal imbalance, or transcriptional reprogramming, rather than an immediate auxin response. Therefore, the observed gene expression profiles may not represent canonical auxin signaling but rather reflect prolonged adaptive effects.

These findings suggest that wheat leaves and roots exhibit differential transcriptional adjustments to the same concentration of auxin. Such differences may arise from the specific physiological roles and developmental needs of different tissues. For instance, as the primary photosynthetic organ, leaves may require more rapid and sensitive transcriptional reprogramming to auxin to modulate morphology and function. In contrast, roots, which are essential for water and nutrient uptake, may prioritize auxin metabolism and transport to maintain normal physiological activities. This observation is consistent with previous research showing that different plant tissues display distinct transcriptional profiles under the same auxin concentration [29]. In this study, *TaPBL7-2B* was identified as a member of the receptor-like cytoplasmic kinase (RLCK) subfamily within the receptor-like kinase (RLK) superfamily. A defining structural feature of RLCKs, including *TaPBL7-2B*, is the absence of an extracellular ligand-binding domain while retaining a highly conserved intracellular kinase domain homologous to that of canonical RLKs [30]. This unique architecture allows RLCKs to bypass extracellular ligand recognition and instead directly interact with membrane-bound receptors or upstream RLKs, potentially facilitating rapid modulation of auxin-mediated physiological processes. Moreover, given the compartmentalized nature of auxin transport and signaling, and the fact that RLCKs often serve as signal integration hubs, it is plausible that *TaPBL7-2B* may exert different regulatory roles depending on the cell type. For instance, in root tissues where directional auxin flow is tightly controlled, *TaPBL7-2B* might modulate PIN transporter activity or downstream signaling sensitivity to auxin, whereas in mesophyll or epidermal cells of leaves, its function may involve feedback suppression of auxin-responsive genes such as *EXPANSINs*. Further investigation using cell-specific expression or localization analyses will be essential to confirm this hypothesis. Overall, the distinct transcriptional profiles observed between wheat leaves and roots following prolonged auxin treatment may reflect tissue-specific regulatory adaptations rather than classical signal-induced responses.

### 4.2. Receptor-like Kinases as Potential Mediators of Auxin-Associated Regulatory Pathways

This study identified several hub genes through Weighted Gene Co-expression Network Analysis (WGCNA) that are involved in diverse biological processes, including protein kinase regulation, transcription factor activity, metabolic processes, and stress response regulation. Among them, plant receptor-like kinases (RLKs), as a class of important signal transduction proteins located on the plasma membrane, drew particular attention due to their broad roles in plant growth and development. Previous studies have shown complex interactions between transcription factors (TFs) and RLKs. These include TFs directly regulating RLK gene expression, RLK-mediated phosphorylation modulating the transcriptional activity of TFs, and feedback loops formed between TFs and RLKs. These interactions constitute intricate signaling networks that fine-tune plant development and environmental responses [31]. In addition to developmental roles, some RLKs regulate metabolic processes related to male reproduction. For instance, the receptor-like kinase LePRK2, localized on the plasma membrane of tomato pollen tubes, can sense extracellular growth cues. Antisense suppression or RNA interference of *LePRK2* results in slower pollen tube growth, demonstrating that LePRK2 positively regulates pollen germination and tube elongation [32]. RLKs also play critical roles in plant responses to abiotic and biotic stresses. For example, *LecRK-I.9* in *Arabidopsis thaliana* encodes a lectin receptor kinase that enhances resistance to *Pseudomonas syringae* by modulating the jasmonic acid (JA) signaling pathway and activating JA-responsive defense genes [33]. Under dehydration stress, the expression of *CLE25* is significantly upregulated in roots. The root-derived CLE25 peptide is transported to the leaves, where it is recognized by BARELY ANY MERISTEMS (BAM) receptors, leading to stomatal closure and enhanced drought resistance [34]. Overall, RLKs function as key sensory proteins that regulate plant growth, development, and stress responses via precise signal transduction mechanisms. In this study, we identified *A0A3B6C1X4* (TraesCS2B02G096000), an RLK family member whose expression was significantly induced by auxin treatment in both root and leaf tissues (Figure 5). Given the prolonged IAA treatment duration (7 days), the observed induction likely represents a sustained transcriptional adaptation rather than an immediate signaling response. On the basis of its chromosomal location and homology to known Arabidopsis genes, it was named *TaPBL7-2B*. Notably, *PBL7* has been implicated in the cold stress response in barley and auxin signaling during early somatic embryogenesis in conifers [16,17]. However, the role of RLKs in auxin signaling in wheat remains largely unexplored. The transcriptional induction of *TaPBL7-2B* under long-term auxin treatment, combined with its putative functional domains, suggests its possible involvement in mediating auxin-associated developmental modulation and stress-related adaptations in wheat.

### 4.3. Wheat Auxin-Associated Gene TaPBL7-2B Negatively Regulates Plant Growth and Development

In this study, the full-length coding sequence (CDS) of *TaPBL7-2B* was cloned from the wheat variety Chinese Spring. Its homologous gene in Arabidopsis, *AtPBL7*, was also studied through the identification of a homozygous *pbl7* mutant. *TaPBL7-2B* was then overexpressed in both Arabidopsis wild-type (Col-0) and *pbl7* mutant backgrounds to validate its function. Phenotypic analysis revealed that *TaPBL7-2B* overexpression lines exhibited an auxin-sensitive phenotype, characterized by inhibited primary root elongation and curled leaves. In contrast, the *pbl7* mutant displayed an auxin-insensitive phenotype, with longer primary roots and flatter leaves. Notably, overexpression of *TaPBL7-2B* in the *pbl7* mutant partially restored auxin sensitivity. These results suggest that *TaPBL7-2B* is an auxin-associated gene that plays a negative regulatory role in plant growth and development. To explore the potential regulatory basis underlying this regulation, transcriptome analysis was performed in *Arabidopsis* overexpression and mutant lines. Differentially expressed genes (DEGs) were significantly enriched in the mitogen-activated protein kinase (MAPK) signaling pathway. The MAPK cascade is a highly conserved eukaryotic signaling module known to act downstream of receptor-like kinases (RLKs), including in plant developmental and stress response pathways [35]. Moreover, both loss-of-function *AtPBL7* and overexpression of *TaPBL7-2B* in Arabidopsis significantly influenced the expression of genes in the auxin signaling pathway. These findings suggest that *TaPBL7-2B*, as a receptor-like kinase, may regulate plant development through both the MAPK cascade and auxin-associated signaling. To further assess the role of *TaPBL7-2B* in wheat, its expression was examined in six wheat varieties with differing auxin sensitivities under both control conditions and 50 μM IAA treatment. The results revealed a potential correlation between *TaPBL7-2B* expression levels and auxin sensitivity in wheat seedlings. This observation supports the involvement of *TaPBL7-2B* in modulating auxin-related responses in wheat, likely through sustained regulatory effects rather than rapid signal transduction.

## 5. Conclusions

This study investigated how different concentrations of indole-3-acetic acid (IAA) influence the growth and development of wheat seedlings, and identified key hub genes involved in this process. Among them, the receptor-like kinase *TaPBL7-2B* was characterized as a crucial factor involved in auxin-associated signaling and transcriptional regulation. The loss-of-function mutant of its homolog (*AtPBL7*) in Arabidopsis displayed auxin-insensitive phenotypes, such as increased leaf size and elongated primary roots, highlighting its conserved role in auxin-associated signaling. In wheat, a positive correlation was observed between *TaPBL7-2B* expression levels and auxin sensitivity across different varieties, suggesting its potential role in mediating auxin-induced phenotypic variation. RNA-Seq analysis further indicated that *TaPBL7-2B* may modulate auxin-associated signaling by influencing gene expression in hormone signaling, MAPK cascades, and stress-related pathways. Collectively, these findings deepen our understanding of hormone signal transduction in wheat and highlight *TaPBL7-2B* as a promising candidate gene for improving plant architecture and stress resistance, ultimately enhancing yield and grain quality.

## Data Availability

All relevant data are within the article and its Supplementary Files.

## Supplementary Materials

The following supporting information can be downloaded at https://www.mdpi.com/article/10.3390/plants14152277/s1. Table S1: Primers used in this study.
