# Supplementary material for: Identification of Auxin-Associated Genes in Wheat Through Comparative Transcriptome Analysis and Validation of the Candidate Receptor-like Kinase Gene TaPBL7-2B in Arabidopsis
Source: Plants (Basel). 2025 Jul 24;14(15):2277. doi: 10.3390/plants14152277 (PMC12348988; doi:10.3390/plants14152277)
Supplement: Supplementary file 1 [file plants-14-02277-s001.zip › plants-3758674-supplementary.pdf]

Table S1. Primers used in this study

Name	Primer (5′-3′)
**Primers for verifying T-DNA insertion mutants**	
SALK_114130LP	AGCCGCTGTCTACAAATGAAG
SALK_114130RP	GAAATTTAAATTCCATGGGGG
LBb1.3	ATTTTGCCGATTTCGGAAC
**Primers for cloning and constructing *TaPBL7-2B***	
TaPBL7-2B-F	GACGCGGAGCACACTCAAAC
TaPBL7-2B-R	TCACTCGCTTCCTCCTCTTTG
Adaptor-F	CACTGTTGATACATATG
Adaptor-R	CTTCATCTTCATAAGAGCTC
**Primers for qRT-PCR**	
AtActin-F	TCTTGTTCCAGCCCTCGTTT
AtActin-R	TCTCGTGGATTCCAGCAGCT
TaActin-F	CTCCCTCACAACAACCGC
TaActin-R	TACCAGGAACTTCCATACCAAC
AtPBL7-F	ATCGAGTGGGAGGAATAAGA
AtPBL7-R	CTGAAGTTTCTAGTGGCAGT
TaPBL7-2B-F	GATCCTTCCCCAGATAAACC
TaPBL7-2B-R	CGTATGTACCCATCACTCTG
TraesCS5A02G365300-F	GCCAAGCTGATCGGGGACA
TraesCS5A02G365300-R	ACCAGAGCTTCACGCAGTAACG
TraesCS2A02G545800-F	GCACTGCCGCTTTGGGTTTG
TraesCS2A02G545800-R	ATTGCTTCTCCCCTTGGCGG
TraesCS6D02G164200-F	AGAGGCCGGTGATGTCTGAAG
TraesCS6D02G164200-R	GCACCGTCTCGCTCGAATC
TraesCS7B02G071100-F	CCCCGTTTCTCCTCTGTTATCTG
TraesCS7B02G071100-R	TGTTCTCTTCAGCTCTACCTCCG
TraesCS2B02G562100-F	GGATAACGCCGACGGGTGC
TraesCS2B02G562100-R	TGGCATACGCCCACATGACG
TraesCS4B02G343200-F	GAGAGGGGAGCTGGCTTACG
TraesCS4B02G343200-R	CCGTGTCATAACTCCCCGCT
TraesCS1B02G283400-F	GCTTTCTTCGCTTGGTTTGGC
TraesCS1B02G283400-R	GAGGTTGGCCTGGAAAACGC
